# Lessons Learned from a Quality Improvement Initiative: Adverse Childhood Experiences Screening in a Pediatric Clinic

**DOI:** 10.1097/pq9.0000000000000482

**Published:** 2021-12-15

**Authors:** Molly M. Crenshaw, Caitlyn R. Owens, Carrie Dow-Smith, Casey Olm-Shipman, Rasheeda T. Monroe

**Affiliations:** From the * University of North Carolina School of Medicine, Institute for Healthcare Quality Improvement; † WakeMed Health and Hospitals Pediatric Primary Care; ‡ Department of Psychology, North Carolina State University.

## Abstract

Universal screening for adverse childhood experiences (ACEs) is recommended by the American Academy of Pediatrics due to downstream health risks. However, widespread screening practices have not been adopted.

**Methods:**

We used quality improvement methods to establish ACEs screening in a busy pediatric clinic that serves primarily Medicaid-insured and Spanish-speaking patients. The final Plan-Do-Study-Act cycle included the screening of both the patient and his/her caregiver(s). ACEs scores were a process measure; balancing measures were the average time to screen, the number of referrals generated, and qualitative caregiver reception.

**Results:**

We screened 232 families, and the process maintained a ≥ 80% completion rate of ACEs screening for 1-month-old children and their caregivers during the final 10 weeks. 23% of caregivers had an ACEs score ≥ 4; overall, 6% were referred for further resources. The average time to discuss the screen was 86.78 seconds. The general caregiver reception was gratitude; 2% refused screening.

**Conclusion:**

This study demonstrates the feasibility of initiating ACEs screening of 1 age group and their caregivers using quality improvement methods.

## Abstract 2

Supplemental Digital Content is available in the text.

## INTRODUCTION

In 1998, Felitti and colleagues published a study showing associations between adverse childhood experiences (ACEs) and downstream negative health outcomes, including chronic disease and premature mortality.^1^ These adverse experiences include childhood abuse, neglect, and household dysfunction (eg, substance use and domestic violence). A recent meta-analysis estimated that in North America, the annual cost attributed to ACEs was approximately $748 billion.^2^ The American Academy of Pediatrics published a call to action in 2011 aimed at increasing pediatricians’ awareness of toxic stress and ACEs, yet pediatricians have been slow to adopt and implement ACEs screening.^3–5^

Pediatricians surveyed regarding obstacles to ACEs screening report concerns regarding the general feasibility of ACEs screening in a busy practice, caregiver resistance, the time needed to administer screening, lack of confidence in discussing ACEs, and lack of interventions to address positive screens.^5–7^ A recent qualitative study that examined caregivers’ responses to ACEs screening found that caregivers accept and appreciate the screen and believed it could help connect their family to needed services.^8^ The clinic in which this study was conducted was awarded a grant to develop Project LAUNCH (Linking Actions for Unmet Needs in Children’s Health), which aims to improve social-emotional health. The integrated mental health professionals on the LAUNCH team offer the following: evidence-based caregiving support during well-child visits, short-term therapy within the clinic, and connection to needed services after ACEs screening.

The purpose of this work was to effectively and efficiently incorporate caregiver and child ACEs screening for 1 age group into a primary care pediatric clinic using a quality improvement model, with a primary goal to achieve ≥ 75% screen completion of eligible patients.

## METHODS

### Context

We conducted the current study at an urban, hospital-based outpatient pediatric clinic. Eight physicians and additional rotating residents serve approximately 7600 unique patients per year; 90% of them have Medicaid, and 50% are Spanish-speaking. The LAUNCH team, 2 physicians, and a medical student pursued integrating ACEs screening. A total of 267 caregivers and 199 children received screening for ACEs during this study. Our institutional review board reviewed and approved this study.

### Project Phases

The project occurred between November 2018 and February 2020. The clinic adopted the Center for Youth Wellness Adverse Childhood Experiences Questionnaire for the screening tool, cited as a preferred screening method.^9,10^ We used the de-identified version of the screen so caregivers disclosed only the number of ACEs instead of identifying specific ACEs they had experienced. The team made 3 minor tool changes to improve clarity (**see document, Supplemental Digital Content 1,** which shows the final screen used.”^9^, http://links.lww.com/PQ9/A321).

**Fig. 1.**
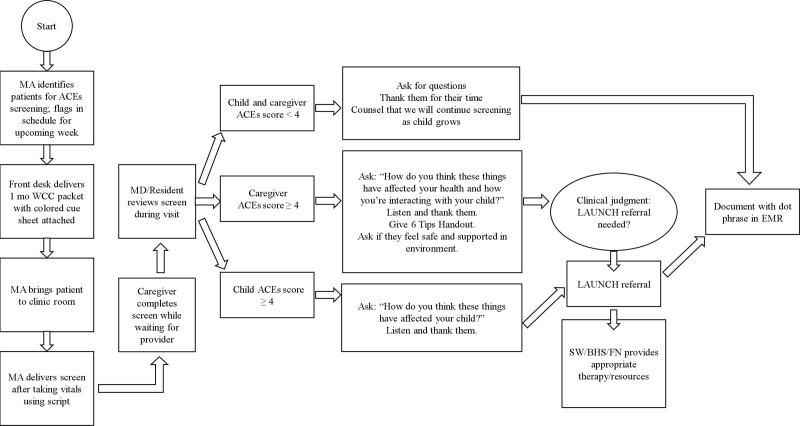
Final process map for embedding ACEs screening into clinic work flow. This was the process used during PDSA cycle 9. WCC, well child check; MD, medical doctor; LAUNCH, Linking Actions for Unmet Needs in Children’s Health; SW, social worker; BHS, behavioral health specialist; FN, family navigator.

The project was divided into 3 phases: Pilot 1, Pilot 2, and the Implementation Phase (Table 1). Baseline data were not available because ACEs screening was not in place before this project. Iterative process changes were made via Plan-Do-Study-Act (PDSA) cycles.^11^ A key driver diagram was created before implementation (see document, Supplemental Digital Content 2, which shows the key driver diagram created before the project started, http://links.lww.com/PQ9/A321). The secondary drivers identified as targeted changes to test were 4-fold: developing efficient and clear explanations of the screen, who administers screening, screened age groups, and developing buy-in from clinic staff. The final clinic flow process map and the script for delivering the screen is described in Figure 1.

**Table 1.** Timeline and Details of PDSA Cycles

Phase (Dates)	PDSA Cycle	No. Physicians Participating	Screen Administrator	Participants	Data Collected
Pilot 1 (11/19/18–2/1/19)	1	2	Medical student	Parent/child (0- to 5-year-olds)	1. Percent complete 2. ACEs scores 3. Qualitative parent reception
	2*	8	Front desk staff		
Pilot 2 (9/18/19–10/23/19)	3	2	Medical student	Parent (0- to 1-year-olds)	1. Percent complete 2. ACEs scores 3. Time to deliver screen 4. Time to discuss screen 5. Referrals 6. Qualitative parent reception
	4	2	MA		
	5	2	Medical Student	Parent/child (0- to 1-year-olds)	
	6	2	MA		
Implementation (10/24/19–2/13/20)	7	8	MA	Parent/child (0- to 1-month-olds)	1. Percent complete 2. ACEs scores 3. Referrals 4. Qualitative parent reception
	8	8	MA		
	9	8	MA		

*Only cycle in which written delivery method was used.

*Pilot 1.* During the small pilot that began PDSA cycle 1, patients of only 2 physicians underwent ACEs screening on a day the screen administrator was present in the clinic if they met the following criteria: aged 0–5 years, arrived for their well-child check, and spoke Spanish or English. Each person who administered the verbal screen throughout the project spoke both English and Spanish. If there was a score of 4 or higher for the caregiver and/or child, the medical student notified the physician, and a LAUNCH referral was made. In PDSA Cycle 2, all 8 physicians participated, significantly increasing the number of screened patients, although only on days when a screen administrator was present. To address long-term feasibility and efficiency in rooming patients, caregivers received a typed letter describing the purpose and procedure instead of a verbal screen delivery; caregivers completed the written screen in the waiting room. The physician discussed the screen in the patient’s room.

*Pilot 2.* Data from Pilot 1 informed significant changes for Pilot 2 because the written letter was not a successful change in the clinic. Thus, the clinic returned to verbal delivery of the screen. The 2 original physicians participated with a narrower age range (0–1 year) to decrease patient numbers as adjustments were made. Pilot 2 began with an “ACEs Week” to increase buy-in from all staff. Staff and physicians watched a documentary about ACEs followed by a discussion and educational displays within the clinic. After that, if the caregiver ACEs score was 4 or higher, they received a handout called “6 Tips to Protect and Heal From Toxic Stress.”^12^ The staff also asked the following question based upon Felitti’s recent publication: “How do you think these experiences have impacted your health today and how do you interact with your child?”^13^ Then, using shared decision-making, the patient and physician reached an agreement regarding whether a LAUNCH referral was indicated for behavioral health services. In PDSA Cycle 5, with successful processes underway for caregivers, patients were also screened to establish their baseline score. If the child’s ACEs score was 4 or more, a LAUNCH referral was made. The “6 Tips to Protect and Heal From Toxic Stress” was delivered, and the caregiver was asked if the child was safe in his/her current environment.^12^ In PDSA Cycle 6, meetings were held with staff and physicians to understand the screening barriers and identify opportunities to optimize the process.

*Implementation Phase.* During the Implementation Phase, all 8 physicians participated, and the eligible age-range narrowed further to 0- to 1-month-olds. PDSA Cycle 8 entailed flagging eligible patients in the electronic medical record (EMR) schedule to identify patients eligible for screening. In PDSA Cycle 9, to further “flag” eligible patients and their caregivers, a bright yellow paper was placed on the front of the 1-month-old WCC packets as a reminder for staff to administer the ACEs screen.

### Measures

The primary measure, percent completed ACEs screens, was calculated by dividing the number of completed screens by the number of families eligible for screening. A secondary goal was to address perceived barriers to ACEs screening. The average ACEs score throughout each PDSA cycle was a process measure. Balancing measures were average time to administer the screen as a function of screen administrator [medical student versus medical assistant (MA)], the average time of physician-caregiver discussion of the screen during the visit, the number of referrals generated by ACEs screening, and caregivers’ perceptions of the ACEs screen. De-identified ACEs scores were captured from the screening sheet or EMR documentation. The medical student and MA measured the times to deliver (explaining ACEs and screening instructions to caregiver) and discuss the screen (physician time to discuss results). Novel LAUNCH referrals resulting from ACEs screening were collected weekly from chart review. The physicians and staff collected caregiver quotes during conversations about ACEs to provide qualitative data about caregiver response.

### Analyses

QI Macros SPC Software for Excel developed by Jay Arthur was used to create control charts.^14^ Standard SPC rules were applied to detect special cause variation. Control limits were set at 3 standard deviations (per convention).^15^ During Pilot 1, single tests of change were employed to identify and develop the necessary infrastructure for sustained implementation of ACEs screening. Once this infrastructure was established, weekly ACEs screening rates were measured beginning with Pilot 2, which served as the baseline. All other analyses were run in SPSS (version 26). An independent samples *t* test was run to examine differences in mean ACEs scores between PDSA Cycles 2 and 9, taking into account an unequal homogeneity of variance as assessed by Levene’s Test for Equality of Variances. An independent samples *t* test was performed to look for mean differences in the time to administer the screen as a function of administrator. We conducted a 1-way ANOVA between-subjects to compare the average discussion time for PDSA Cycles 3, 4, and 5. Emerging themes categorized caregivers’ comments about the screen.

## RESULTS

Pilot 1, Pilot 2, and the Implementation Phase lasted 10, 6, and 17 weeks, respectively, during which 55, 52, and 125 families received screening, respectively.

### Screening Completion Rates

**Fig. 2.**
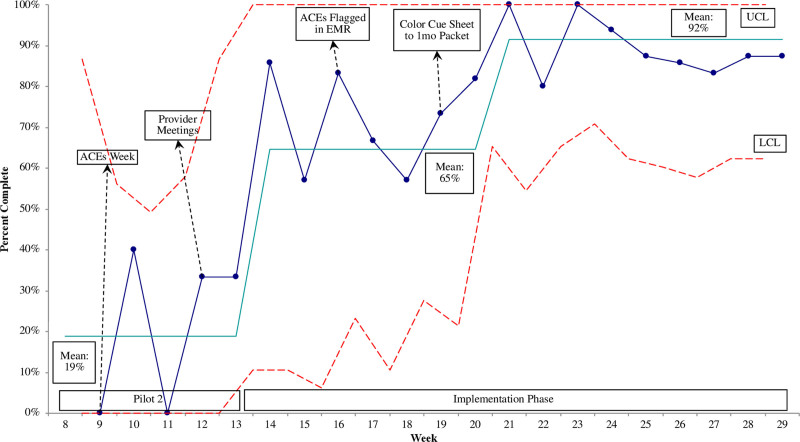
Statistical Process Control Chart: P Chart of ACEs screening completion rate for 1-month-old patients (n = 126) starting with Pilot 2. *x* axis: project week number. *y* axis: weekly percent completion of ACEs screening (the number completed screens divided by the number of families eligible for screening). UCL and LCL (dotted lines) are set as 3 standard deviations above and below the mean. Solid straight line is the mean. LCL, lower control limit; UCL, upper control limit.

The screening completion rate ranged from 58% to 100% (Table 2). Of the 284 intended screens, 58 (20%) screens were incomplete. Reasons for incomplete screens included staff forgetting to deliver the screen, physicians failing to document, caregivers misunderstanding instructions, misplaced screens, and caregiver refusals. There were 5 total refusals, making up 9% of incomplete screens and 2% of total families screened. The completion rate was > 80% for the final 10 weeks of data collection (Fig. 2).

**Table 2.** Number of Screens and Mean Adverse Childhood Experiences Scores for each PDSA Cycle

PDSA Cycle	Parents		Children		Percent of Goal Screened
	Screens Complete	Mean (SD)	Screens Complete	Mean (SD)	
1	25	3.60 (4.76)	21	1.05 (2.50)	95%
2	43	0.51 (1.10)	34	0.12 (0.41)	58%
3	18	4.56 (3.57)	0	NA	100%
4	18	3.30 (4.27)	1	2.00 (NA)	84%
5	19	3.05 (4.20)	15	0.27 (0.80)	88%
6	12	1.00 (1.28)	9	0.11 (0.33)	90%
7	13	1.00 (1.53)	11	0.09 (0.30)	69%
8	17	2.18 (2.48)	17	0.18 (0.53)	68%
9	102	2.40 (3.42)	91	0.32 (0.76)	88%

### ACEs Scores

**Fig. 3.**
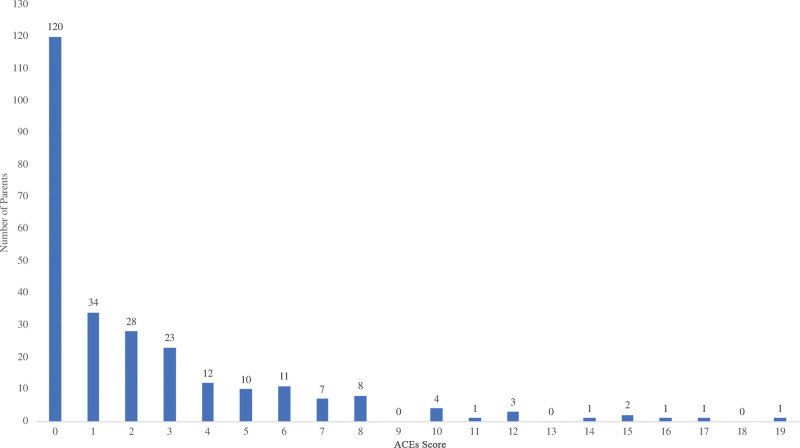
Frequency of caregiver ACEs scores (N = 267).

**Fig. 4.**
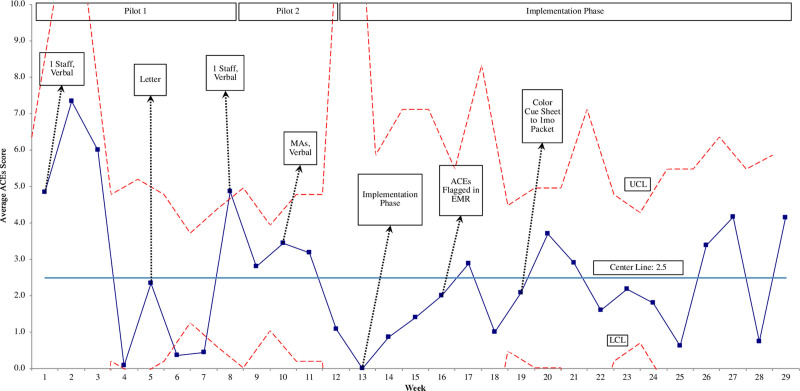
Statistical Process Control Chart: X-bar Chart of Caregiver ACEs score (N = 267) by week. *x* axis: project week number. *y* axis: average weekly caregiver ACEs scores. UCL and LCL (dotted lines) are set as 3 standard deviations above and below the mean. The solid straight line is the mean. “1 staff” indicates 1 dedicated person whose only clinic responsibility was to deliver the screen. “Verbal” and “Letter” indicate how the screen was administered. LCL, lower control limit; UCL, upper control limit.

The overall average caregiver ACEs score was 2.32 (SD = 3.42). ACEs scores of 4 or greater occurred in 23% of caregivers screened (Fig. 3). Scores in Cycle 9 (M = 2.40, SD = 3.42), which had a verbal delivery method, were significantly higher than scores in Cycle 2 (M = 0.51, SD = 1.10), the only cycle with a written delivery method (*P <* 0.001). The average caregiver ACEs score for each week demonstrated decreased variation over time (Fig. 4).

### Time to Deliver and Discuss Screen

The average time to administer the caregiver-only screen was 68.06 seconds (SD = 29.88), and the average time to administer the caregiver and child screen was 92.44 seconds (SD = 21.57). There was no significant difference in time when the screen was administered by the medical student (M = 78.69, SD = 33.16) versus the MA (M = 58.85, SD = 24.14) (*P* = 0.079). The average time to discuss all screens was 86.78 seconds (SD = 79.59) with a range of 0–420 seconds. Six of the 42 timed discussions were longer than 2 minutes.

### Referrals

There were 12 (6%, n = 199) novel LAUNCH referrals during Pilot 2 and the Implementation Phase. Of the 50 (25%, n = 199) caregivers reporting an ACEs score of 4 or greater, 8 (16%, n = 50) were deemed clinically appropriate for a new LAUNCH referral. Many caregivers declined because they reported feeling well-supported. Five (3%, n = 149) additional referrals were made for caregivers who reported an ACEs score of < 4.

### Qualitative Caregiver Reception of Screen

Five themes emerged from caregiver feedback: gratitude, the notion these events were in their past, an acknowledgment that these events have had an impact on both their health and the health of their child, a desire to seek professional help to process these experiences, and the idea that these experiences have made them stronger. Comments include:

“Thank you for taking the time. It means you care.”

“I have gone to therapy about these things, and now I am just enjoying my life.”

“[The screen] made me realize how important it was to raise my kid better than how I was raised. You know, break the cycle.”

“I hope to teach my son not to bully others but to love everyone. I was bullied as a kid.”

## DISCUSSION

Using Improvement Science methodology, we embedded ACEs screening of 1-month-old children and their caregivers into a busy pediatric clinic.^11^ There was at least an 80% completion rate during the final 10 weeks of data collection, higher than reported in a similar study.^16^ While ACEs screening does not replace acute psychosocial needs assessments that ensure the child’s safety, ACEs screening can contribute to a healthier tomorrow for our children and families. With ACEs screening, providers have a conduit to initiate a conversation within the clinic about toxic stress, to empower parents to help their child build resilience, and to offer strategies to decrease the effects of toxic stress both for the child and the parent.^8,12,17^

### Lessons Learned

*Sustainable screening processes can be established.* The sustained completion rate demonstrates that ACEs screening in 1 age group is feasible in a busy primary care clinic serving a vulnerable population. Strategies such as choosing the 1-month-old WCC because there are relatively few forms requiring completion probably allowed for meaningful caregiver engagement with the ACEs screen. Additionally, we believe EMR flagging and brightly colored paper as visual cues increased successful screenings. Following recent literature, buy-in was relentlessly pursued from all clinic staff to enable a culture shift in prioritizing ACEs screening, including incomplete screens and lack of documentation experienced, which was observed early in the project.^18^ Buy-in strategies included 1-on-1 provider coaching during on-boarding, an “ACEs Week,” and providing staff and physicians updates and opportunities to give feedback throughout the implementation.

*Face-to-face delivery is important.* Literature shows that physicians feel ill-equipped to deliver and discuss ACEs information with families.^5,6^ Our data suggest that caregivers were significantly more willing to disclose, as measured by increasing average ACEs scores, when a verbal delivery method was used instead of a written delivery. These findings are consistent with previous literature.^19^ Twenty-three percent of caregivers screened in our study reported an ACEs score of 4 or more, when compared with 16% of the US population.^20^ This suggests that (1) our population is at higher risk than the general population, (2) the delivery method used makes them feel safer to disclose, and/or (3) this population is more comfortable disclosing than the general population. Other reasons for this finding could be sample size, lack of clarity and brevity in the written letter, or that our clinic’s literacy level rendered a written letter delivery method ineffective.

*Time added to visit: Less than 2 minutes per patient*. The time to deliver data suggests that delivery is a skill that can be efficiently learned and embedded within an MA’s clinic duties. It is patient-centered for the MAs to provide the screen because they usually have developed an element of rapport with the patients throughout the rooming process. On average, it took 92 seconds to deliver the child and caregiver screens. Also our assumption was that ACEs screen delivery did not add to the patient visit time because it was administered when the patient was awaiting the physician.

However, time added for physician discussion of the screen potentially adds to visit duration, a concern voiced by physicians in this clinic and reported in the literature.^5–7^ The average physician time to discuss the screen was 87 seconds, although these data were limited by sample size. While a 2-minute addition to each visit is significant, it is also possible that the screen helps streamline the conversation, ultimately decreasing visit times, but more studies are needed to determine how this affects total visit time. One study found that screening for ACEs did not affect average clinic visit duration.^21^

*Physicians infrequently referred with a positive screen*. One of the biggest concerns in the clinic was that screening for ACEs would result in an unrealistic number of referrals to the behavioral health team. However, the referral rate was only 6% of those screened for ACEs. Referral rate could be low because physicians either do not see the benefit of referral or find referral burdensome. Possibly, the script provided a level of comfort that reduced the perceived need for additional behavioral health intervention. Learning from postpartum depression screening, it could be that the act of screening alone is a form of intervention.^22^ Additionally, another plausible reason is that families in this clinic are already connected to behavioral health services, allowing ACEs screening to serve as an educational platform to discuss the negative effects of toxic stress. This clinic already had established behavioral health services before this quality improvement project, which may limit the generalizability of this study.

*Caregiver gratitude.* Caregivers appreciated the clinic’s willingness to ask questions about their past. One caregiver noted that they felt the clinic better understood his family as a result of the screen. One of the perceived barriers held by pediatricians is that caregivers will negatively perceive the screen.^5,6^ Findings in this and other studies suggest that caregivers are receptive to screening and deem it important in the pediatric clinic.^7,8,23^ Of the 5 refusals, only 1 caregiver refused because they seemed distrustful of the screen, and the other 4 were due to clinic logistics not conducive to a successful screen completion. Asking for feedback in person during the visit potentially introduced biases into our responses, resulting in more positively skewed responses, although previous literature also demonstrates generally positive caregiver reception.^8^

There is controversy about whether caregiver ACEs screening should be conducted in a pediatric clinic due to potential triggering or lack of positive screen interventions.^24–26^ We did not experience this. Caregivers showed that they learned about ACEs through the screening process. Recent literature suggests that children of caregivers with high ACEs scores are at an increased risk of missing preventive healthcare visits and delayed acquisition of developmental milestones, suggesting that ACEs are intergenerational. Thus, an approach targeting both the caregiver and child is needed to address ACEs adequately.^18,22,27^ Screening caregivers allowed us to educate about ACEs, discuss resilience factors, and ultimately empower caregivers to mitigate the risks of toxic stress for both themselves and their child. Furthermore, ACEs screening identified patients at risk for developing new ACEs and ACEs sequelae. Early behavioral health referrals may mitigate such sequelae. This anticipatory guidance aimed at developing strategies to mitigate the effects of toxic stress is an intervention both feasible for the primary care pediatrician and supported by the literature when addressing a high ACEs score in clinic.^17^

There are several limitations to our study. First, it was conducted at a single center and our interventions may not work in other centers, although most of the changes we made are fundamental change concepts that we believe should be effective in other settings. As discussed above, the behavioral health services already established in the clinic allowed for more accessible interventions when a patient had a high ACEs score. Second, our work was conducted in a single age group (1-month-olds). Because the 1-month-old visit often has fewer items that need discussion and the physical examination is relatively brief compared with that in older age groups, care providers may have been more receptive to adding this additional screen to the visit. The sample size of our study was another limitation specifically for the time data. With a larger sample size, more accurate conclusions may be reached regarding the amount of time ACEs screening adds or saves during a clinic visit. Finally, there were limitations in which the quotes were elicited because they occurred during a clinical visit, and they were elicited by any member of the team, both of which likely introduced biases into the responses. However, our results are consistent with other literature demonstrating generally positive caregiver reception of ACEs screening.^8^

### Next Steps

ACEs screening sustainability for 1-month-olds and their caregivers will be ensured through ongoing performance review of data with clinic staff and physicians. Over time, additional age groups will be included in the screening with concurrent PDSA cycles to inform the development of streamlined processes.

## CONCLUSIONS

This study successfully reached a sustained ACEs screening completion rate of greater than 80%, demonstrating that ACEs screening in 1 infant age group is feasible in a busy primary care clinic serving a vulnerable population. ACEs screening can start the conversation about toxic stress and build trust with caregivers.^8^ We believe that for every child to reach their full potential, screening tools such as the ACEs screen are key elements that when used effectively, with the correct training for providers, and within the context of a trauma-informed community, we can improve the community health to that which we all aspire for our patients and families.^28–30^ In the words of ACEs research giant Felitti, “*Asking*...coupled with *listening* and implicitly accepting the person who had just shared his or her dark secrets, is a powerful form of *doing*.” ^13(p5)^

## DISCLOSURE

The authors have no financial interest in relation to the content of this manuscript.

## ACKNOWLEDGMENTS

The authors thank the physicians of Raleigh Campus Pediatric Primary Care WakeMed Physician Practices. Additional thanks to The University of North Carolina’s Institute for Healthcare Quality Improvement for facilitating the study.

## Supplementary Material


