# Supplementary material for: Lessons Learned from a Quality Improvement Initiative: Adverse Childhood Experiences Screening in a Pediatric Clinic
Source: Pediatr Qual Saf. 2021 Dec 15;6(6):e482. doi: 10.1097/pq9.0000000000000482 (PMC8678025; doi:10.1097/pq9.0000000000000482)
Supplement: Supplementary file 1 [file pqs-6-e482-s001.pdf]

CAREGIVER

CYW Adverse Childhood Experiences Questionnaire (ACE-Q) Self-Report

To be completed by Patient

Today's Date: ______________________________

Your Name: ____ Date of birth: ______________________________

Many children experience stressful life events that can affect their health and development. The results from this questionnaire will assist your doctor in assessing your health and determining guidance. Please read the statements below. Count the number of statements that apply to you and write the total number in the box provided.

Please DO NOT mark or indicate which specific statements apply to you.

1) Of the statements in section 1, HOW MANY apply to you? Write the total number in the box.

Section 1. *At any point since you were born... and when you were 18 years old*

- Your parents or guardians were separated or divorced, never lived together, or a romantic partner moved in or out
- You lived with a household member who served time in jail or prison
- You lived with a household member who was depressed, mentally ill or attempted suicide
- You saw or heard household members hurt or threaten to hurt each other
- A household member swore at, insulted, humiliated, or put you down in a way that scared you OR a household member acted in a way that made you afraid that you might be physically hurt
- Someone touched your private parts or asked you to touch their private parts in a sexual way that was unwanted, against your will, or made you feel uncomfortable
- More than once, you went without food, clothing, a place to live, or had no one to protect you
- Someone pushed, grabbed, slapped or threw something at you OR you were hit so hard that you were injured or had marks
- You lived with someone who had a problem with drinking or using drugs
- You often felt unsupported, unloved and/or unprotected

2) Of the statements in section 2, HOW MANY apply to you? Write the total number in the box.

Section 2. *At any point since you were born... and when you were 18 years old*

- You have been in foster care
- You have experienced harassment or bullying at school
- You have lived with a parent or guardian who died, had a serious physical illness, or disability
- You have been separated from your primary caregiver through deportation or immigration
- You have had a serious medical procedure or life threatening illness
- You have often seen or heard violence in the neighborhood or in your school neighborhood
- You have been detained, arrested or incarcerated
- You have often been treated badly because of race, sexual orientation, place of birth, disability or religion
- You have experienced verbal or physical abuse or threats from a romantic partner (i.e. boyfriend or girlfriend)

CHILD

CYW Adverse Childhood Experiences Questionnaire (ACE-Q) Child

To be completed by Parent/Caregiver

Today's Date: ______________________________

Child's Name: ____ *Date of birth:* ______________________________

Your Name: ____ *Relationship to Child:* _________________________

Many children experience stressful life events that can affect their health and wellbeing. The results from this questionnaire will assist your child's doctor in assessing their health and determining guidance. *Please read the statements below. Count the number of statements that apply to your child and write the total number in the box provided.*

Please DO NOT mark or indicate which specific statements apply to your child.

1) Of the statements in Section 1, HOW MANY apply to your child? Write the total number in the box. [ ]

Section 1. *At any point since your child was born…*

- Your child's parents or guardians were separated or divorced, never lived together, or a romantic partner moved in or out
- Your child lived with a household member who served time in jail or prison
- Your child lived with a household member who was depressed, mentally ill or attempted suicide
- Your child saw or heard household members hurt or threaten to hurt each other
- A household member swore at, insulted, humiliated, or put down your child in a way that scared your child OR a household member acted in a way that made your child afraid that s/he might be physically hurt
- Someone touched your child's private parts or asked your child to touch their private parts in a sexual way
- More than once, your child went without food, clothing, a place to live, or had no one to protect her/him
- Someone pushed, grabbed, slapped or threw something at your child OR your child was hit so hard that your child was injured or had marks
- Your child lived with someone who had a problem with drinking or using drugs
- Your child often felt unsupported, unloved and/or unprotected

2) Of the statements in Section 2, HOW MANY apply to your child? Write the total number in the box. [ ]

Section 2. *At any point since your child was born…*

- Your child was in foster care
- Your child experienced harassment or bullying at school
- Your child lived with a parent or guardian who died
- Your child was separated from her/his primary caregiver through deportation or immigration
- Your child had a serious medical procedure or life threatening illness
- Your child often saw or heard violence in the neighborhood or in her/his school neighborhood
- Your child was often treated badly because of race, sexual orientation, place of birth, disability or religion

CYW ACE-Q Child (0-12 yo) © Center for Youth Wellness 2015

Key Driver Diagram

GLOBAL AIM

Integrate ACEs screening into workflow at WakeMed Pediatric Clinic

SMART AIM

Increase ACE screening tool completion rate from 0% to 75% for specific patients and parents in the WakeMed Peds Clinic by April, 2020 and sustain for 6 months and beyond

PRIMARY DRIVERS
(Needed system factors)

INTERVENTIONS
(Changes to test)

Parental understanding of ACE tool concept

1. Create talking points in English, Spanish, Other
2. Train Residents, med students, MDs, MAs, RNs
3. Make someone available for questions
4. Develop buy in from clinic staff: all know talking points
5. Address cultural barriers resulting in hesitancy to disclose?

Data collection

1. **Create central location for collection of survey hard copies**
2. **Standardize who writes note and enters ACEs score**
3. **Simulate ASQ model: who enters those enters ACEs score**
4. **Ensure staff know who needs ACE questionnaire completed and confirm**

Follow up for high ACE scores

1. **Develop resources for LAUNCH referral during visit only**
2. **Ensure overall sufficient clinic resources**
3. **Develop follow up algorithm for family**

SDC.

Final delivery script. Script used by medical assistants to deliver the adverse childhood experiences screen to parents during plan-do-study-act cycles 5, 6, and throughout the Implementation Phase.

"We are screening every child under the age of 12-months and their caregiver for stressful events that happened to both the caregiver and the child before the age of 18-years-old. We know that stressful events that happen early on can affect our health and how we parent today, so this will hopefully allow us to take better care of you and your family. The form is double sided: one for you and one for your child. For each side, we need you to count the number of events that have happened to you and place that number in the box. We do not need to know which ones, just how many. For example, if this one and this one have happened to you before the age of 18 years, place a 2 in the box. Same for the box below. On this side, if this one, this one, and this one have happened to your child, place a 3 in the box. Same for the box below. A provider will be in to briefly discuss during your visit."
